# The intricate relationship of histoplasmosis and sarcoidosis: a case report

**DOI:** 10.1186/1752-1947-8-235

**Published:** 2014-06-27

**Authors:** Poonam Mathur, John J Zurlo, Tonya J Crook

**Affiliations:** 1 Penn State/Milton S. Hershey Medical Center Department of Medicine, 500 University Drive, Hershey PA 17033, Pennsylvania, USA; 2 Penn State/Milton S. Hershey Medical Center Division of Infectious Diseases, Department of Medicine, 500 University Drive, Hershey PA 17033, Pennsylvania, USA

**Keywords:** Histoplasmosis, Immunosuppression, Sarcoidosis, Steroids

## Abstract

**Introduction:**

Histoplasmosis is an endemic mycosis with most cases of clinical illness reported in North and Central America. Rarely, patients develop progressive disseminated histoplasmosis with extrapulmonary manifestations. These infections are fatal if not appropriately treated.

**Case presentation:**

We report a case of progressive disseminated histoplasmosis presenting with fever, progressive dyspnea, and pancytopenia in a 51-year-old Caucasian man who had been treated with chronic steroids for a diagnosis of sarcoidosis made 20 years previously. His presentation was initially mistaken for sarcoidosis but, fortunately, laboratory results showed hematologic abnormalities, and the diagnosis of histoplasmosis was made by bone marrow biopsy.

**Conclusions:**

Sarcoidosis reduces T cell activity, and the addition of steroids for treatment causes further immunosuppression and vulnerability for development of a disseminated infection. The diagnosis of histoplasmosis depends mainly on clinical presentation and host factors. Although there are diagnostic laboratory tests available, clinicians may need to diagnose histoplasmosis by history and physical examination alone and treat empirically, since awaiting *Histoplasma*-specific laboratory results would delay initiation of treatment. Primary care providers, hospitalists, and subspecialists alike should be aware of the overlap in clinical and radiological presentations of sarcoidosis and histoplasmosis, and when and how to pursue diagnostic testing for endemic mycoses, since these infections can be fatal in immunosuppressed patients without appropriate treatment.

## Introduction

*Histoplasma capsulatum* is found worldwide, but histoplasmosis as a clinical illness is reported most frequently in North and Central America [1]. In the USA, clinical infection is usually associated with areas spanning the Ohio and Mississippi River Valleys [2,3]. Although histoplasmosis is the most common endemic mycoses in the USA [2], it is often overlooked as an infectious etiology in areas outside the Midwest. Most cases of histoplasmosis present as a self-limited acute pulmonary infection. A minority of patients develops extrapulmonary infection, or progressive disseminated histoplasmosis (PDH), usually after initiation of immunosuppressive therapy [2]. PDH may occur with or without preceding acute pulmonary infection and usually occurs after 7 to 21 days of incubation, with symptoms of fever and malaise manifested by day 14 [2]. Laboratory studies may demonstrate hematologic and hepatic abnormalities. Treatment with amphotericin is needed until defervescence, followed by itraconazole for step-down therapy. Without proper treatment, the disease can be fatal.

## Case presentation

**Figure 1:**
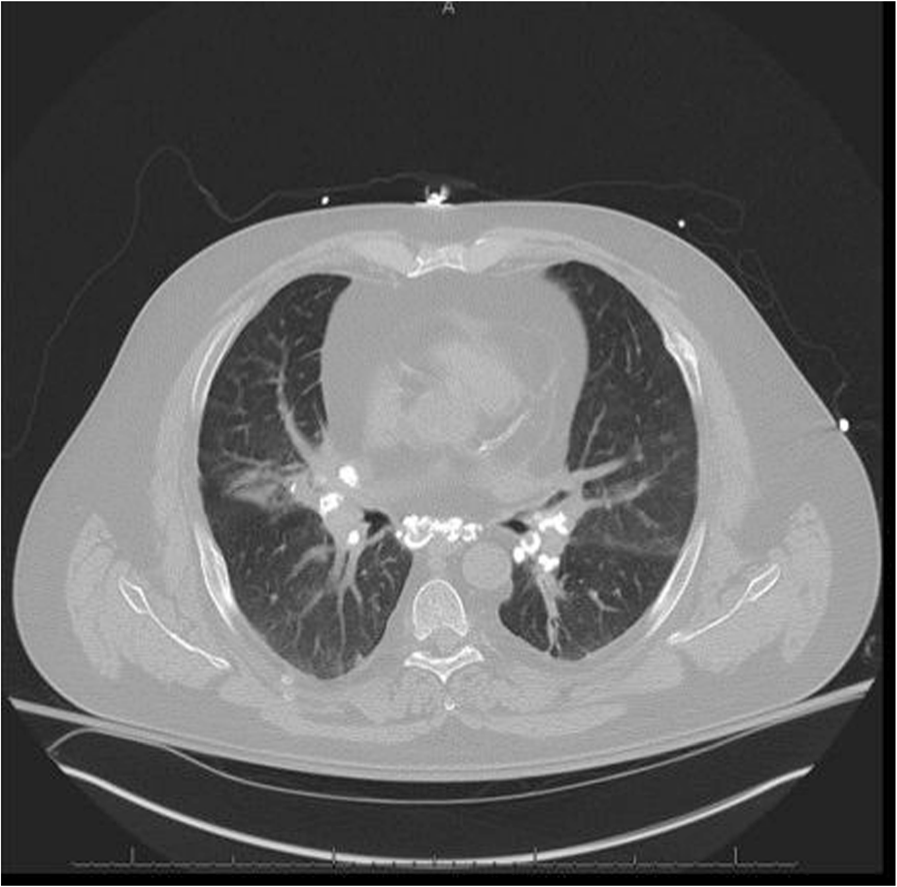
**Noncontrast-computed tomography scan.** This screenshot of the chest computed tomography scan obtained at admission shows pulmonary nodules and calcified mediastinal and hilar lymphadenopathy, which can be found both in sarcoidosis and *Histoplasma* infections.

A 51-year-old Caucasian man with a 20-year history of sarcoidosis was admitted to our hospital with a 1-month history of daily fevers, malaise, and progressive dyspnea. He had been taking prednisone 20mg daily for the past 10 years, and took levofloxacin 750mg daily for 1 week before admission without improvement. He had never smoked cigarettes and had always lived in central Pennsylvania without recent travel history. No sick family members were reported and he had no unusual animal exposures. On examination, he was febrile, tachycardic, hypotensive, and diaphoretic. A lung examination showed fine scattered inspiratory crackles. There was no lymphadenopathy or hepatosplenomegaly. The rest of the examination was unremarkable.Compared to normal values documented 1 month prior, admission laboratory investigations showed a platelet count of 19u/L (normal 150 to 350u/L), hematocrit of 31.5%, white blood cell count of 3.1K/uL (normal 4 to 10.4K/uL), and absolute lymphocyte count of 0.37K/uL (normal 1.0 to 3.4K/uL). The rest of his laboratory tests were within normal limits. Sputum Gram stain and bacterial blood cultures were negative. A noncontrast-computed tomography (CT) scan of his chest showed multiple pulmonary nodules and calcified hilar and mediastinal lymphadenopathy (Figure 1). An initial radiology report advised that fungal infection, such as histoplasmosis, should be included in the differential diagnosis; however, when his medical history of sarcoidosis was revealed, the report was changed to suggest that the imaging findings were due only to sarcoidosis.

**Figure 2:**
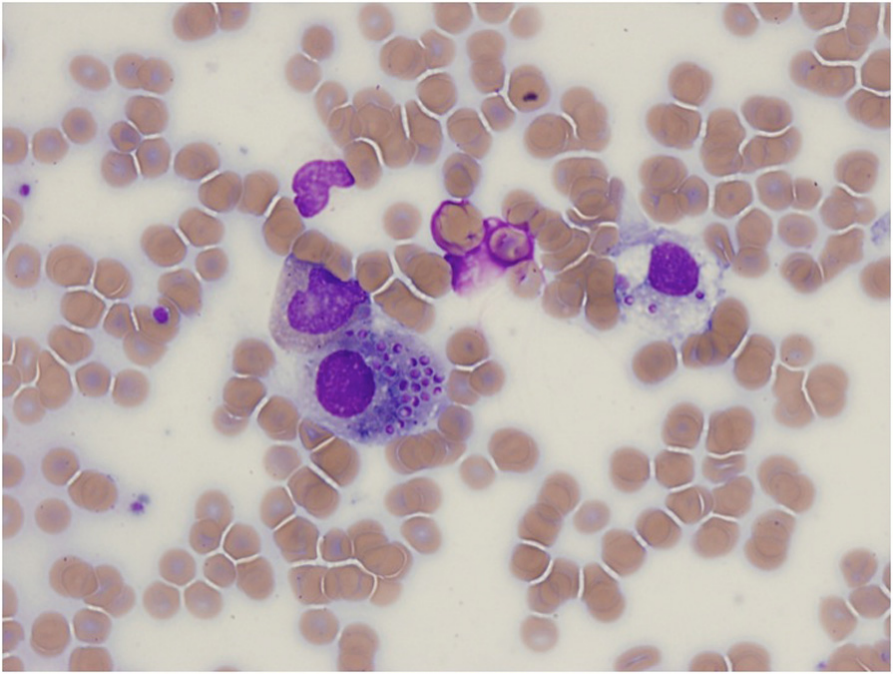
**Bone marrow biopsy.** Wright stain of bone marrow aspirate shows inclusions of *Histoplasma capsulatum* in white blood cells.

Due to his pancytopenia, a bone marrow biopsy was performed, showing yeast-like macrophage inclusions (Figure 2). His urine antigen for *Histoplasma* was positive. *Histoplasma capsulatum* grew from admission fungal blood cultures after 6 days and bone marrow fungal cultures became positive after 11 days. He received liposomal amphotericin for 1 week at a daily dose of 5mg/kg, and clinically improved with resolution of fever in 48 hours. He was then changed to oral itraconazole and continued on this medication as an out-patient. A human immunodeficiency virus enzyme-linked immunosorbent assay was negative. He was eventually switched to fluconazole 800mg daily due to intolerance of side effects from itraconazole.

## Discussion

PDH in immunosuppressed patients can be a fatal disease, therefore diagnosis must be made in the appropriate clinical setting so that treatment is expedited. Optimal diagnostic testing depends mainly on clinical manifestations and host factors [1]. Fungal stain of a bone marrow biopsy can be the most rapid method of diagnosis when suspecting disseminated histoplasmosis, but has only 43% sensitivity. Blood and bone marrow culture provide definitive diagnosis and have 85% sensitivity for disseminated infection. Antibody detection can be rapid; however, there are serologic cross-reactions from other fungi, particularly *Blastomyces*. Also, antibody testing has a high false-negative rate in patients with disseminated histoplasmosis. Therefore, blood and urine *Histoplasma* antigen detection, which has a rapid turnaround time and 92% sensitivity for disseminated infection, has become the diagnostic test of choice [1].

The epidemiology of cases of PDH identifies immunosuppression and age greater than 54 as a major risk factor [2]. Superinfection with mycoses in the setting of sarcoidosis has been described in previous case reports [3-6]. Patients with active sarcoidosis already have suppressed systemic cell-mediated immunity due to a reduced number of circulating T cells [4,6]; doses of steroids as low as 10mg of prednisone equivalent per day further exacerbate this immunosuppression, increasing the risk for atypical/opportunistic infections [7]. Previous case reports also demonstrate that endemic mycoses may be mistaken for sarcoidosis [3-6]. Such was seen in our patient, as his initial chest CT scan interpretation suggested fungal infection, but knowledge of the patient’s sarcoidosis history influenced the final interpretation to suggest the CT findings were consistent only with sarcoid disease.

Our patient was diagnosed with sarcoidosis 20 years earlier. We are uncertain how the diagnosis was made and are unclear whether he had caseating or non-caseating granulomas on pathology at the time of diagnosis. This is an important distinction, as the dynamic relationship between sarcoidosis and disseminated fungal infections has been reviewed by several case studies. A report by Wheat *et al*. suggests that histoplasmosis may trigger a chronic inflammatory disease recognized as sarcoidosis [8]. We think it is unlikely that our patient’s initial presentation 20 years prior was due to histoplasmosis because he clinically improved on steroids, but could speculate that he had an initial self-limited *Histoplasma* infection that led to a state of chronic inflammation similar to sarcoidosis.

## Conclusions

Due to its multiorgan involvement, histoplasmosis must be recognized as an opportunistic pathogen in acutely ill patients with chronic immunosuppression. We report this case to demonstrate that when fever or exacerbation of symptoms occur in a patient with a previous diagnosis of sarcoidosis, clinicians must maintain a high index of suspicion for opportunistic infection with endemic mycoses.

## Consent

Written informed consent was obtained from the patient for publication of this case report and accompanying images. A copy of the written consent is available for review by the Editor-in-Chief of this journal.

## Abbreviations

CT: Computed tomography; PDH: Progressive disseminated histoplasmosis.

## Competing interests

The authors declare that they have no competing interests.

## Authors’ contributions

JZ and TC directly cared for the patient, acquired in-patient and out-patient data, and analyzed and interpreted the data. PM and TC contributed to conception and design of the case report, as well as drafting of the manuscript. PM, JZ and TC revised the manuscript crucially for important intellectual content and created the final version to be submitted. All authors read and approved the final manuscript.
